# Lifestyle, genetic susceptibility, and risk of diverticular disease: a prospective cohort study

**DOI:** 10.1097/JS9.0000000000003087

**Published:** 2025-07-18

**Authors:** Chuan-Guo Guo, Jiaxin Wang, Yufan Liu, Wen Yu, Cuicui Shao, Feifei Zhang

**Affiliations:** a Department of Gastroenterology, Beijing Friendship Hospital, Capital Medical University, Beijing, China; b State Key Laboratory of Digestive Health, Beijing, China; c National Clinical Research Center for Digestive Disease, Beijing, China; d Beijing Key Laboratory of Early Gastrointestinal Cancer Medicine and Medical Devices, Beijing, China; e School of Basic Medical Sciences, Capital Medical University, Beijing, China; f Department of Gastroenterology, Laizhou City People’s Hospital, Laizhou, Shandong Province, China; g Department of Gastroenterology, Cangzhou Fourth Hospital (Nanpi County People’s Hospital), Cangzhou, Hebei Province, China; h Center for Digital Health and Artificial Intelligence, Peking University First Hospital, Beijing, China; i National Institute of Health Data Science at Peking University, Peking University Health Science Center, Beijing, China; j Institute of Medical Technology, Peking University Health Science Center, Beijing, China

**Keywords:** diverticular disease, diverticulitis, lifestyle, polygenic risk score

## Abstract

**Background:**

Emerging evidence indicates that diverticular disease is attributed to both environmental and genetic factors. The impact of modifiable lifestyle factors on diverticular disease has not been fully elucidated, particularly regarding the role of genetic predisposition.

**Materials and Methods:**

We performed a prospective cohort study using data from the UK Biobank, which included 472 612 participants. The impacts of 17 lifestyle factors on diverticular disease were evaluated using Cox regression models. Sensitivity analysis was performed with a specific focus on complicated diverticulitis with perforation and abscess. Stratified analyses were performed according to polygenic risk score (PRS) tertiles.

**Results:**

Over a median follow-up of 13.6 years, we identified 23 742 cases of diverticular disease, including 832 cases of complicated diverticulitis. After adjusting for age, sex, BMI, ethnicity, household income, and PRS tertiles, smoking, alcohol, frequent insomnia, sedentary behavior, and tea consumption were associated with an increased risk of diverticular disease. Conversely, intermediate sleep duration, coffee consumption, and a healthy diet were associated with a lower diverticular disease risk. For complicated diverticulitis, smoking and frequent sleeplessness/insomnia remained as significant risk factors. A healthy diet, particularly one rich in fruits and wholegrains, was associated with reduced risk of complicated diverticulitis. The effects of smoking, sleeplessness or insomnia, and consumption of refined grains, processed meats, and unprocessed red meats on diverticular disease may be modified by genetic predisposition.

**Conclusions:**

Adopting an optimal lifestyle is associated with a lower risk of developing diverticular disease, while the impact of certain lifestyle factors may be modified by genetic predisposition.

## Introduction

Diverticular disease is a prevalent gastrointestinal condition that contributes to significant morbidity and considerable healthcare expenditure[1]. In Western and industrialized societies, colonic diverticular disease is one of the leading causes of hospital admissions[2]. Caucasian groups exhibit higher prevalence, while Asian regions display intermediate prevalence (e.g., Korea: 12.1%; Singapore: 20.0%), and it is relatively low in Africa and South Asia[3]. Of the common diverticular diseases, diverticulitis – a condition characterized by inflammation of the diverticulum – can lead to severe complications and recurrences. For example, 13–23% of people with uncomplicated diverticulitis develop recurrence, while this figure increases as high as 40% in those with complicated diverticulitis[4]. In the United States, diverticulitis results in more than 629 000 emergency department visits and 323 000 hospitalizations each year, with associated healthcare costs of around $9 billion[5].

Increasing evidence shows that diverticular disease may be attributed to both environmental and genetic risk factors.^[1,6-10]^ Among the various environmental factors, lifestyle and dietary habits play a significant role in modulating the risk of diverticulitis. For example, modifiable risk factors including obesity, smoking, physical inactivity, and a low-fiber diet have consistently been identified in previous studies[6]. A diet rich in fiber from fruits and cereals is associated with a reduced risk of the condition[11]. Additionally, a high intake of red meat, particularly unprocessed red meat, has been linked to an increased risk of diverticulitis[12]. Genetic studies have also shown that diverticular disease has a heritability of 40–53%[13]. Recent genome-wide association studies have identified 42–150 risk loci related to diverticulitis^[8-10,14]^.

Previous research primarily focused on either environmental or genetic factors, but the modified effect of lifestyle factors by genetic risk on diverticular disease remains unclear. Our study investigated the influence of lifestyle factors and polygenic risk score (PRS) on the likelihood of developing diverticular disease in a large cohort of people in the UK Biobank (UKB). By integrating modifiable lifestyle factors with inherent genetic predispositions, we aimed to enhance the predictive accuracy of diverticular disease risk and inform effective preventative strategies.

## Methods

### Study population

We used data from the UKB, an ongoing prospective cohort study in the UK consisting of more than 500 000 participants aged 40–70 years. The UKB protocols were approved by North West-Haydock Research Ethics Committee (REC reference: 21/NW/0157). Participants signed written informed consent for health-related research. This study was approved by the UKB under application 90018. Participants were excluded if they had been hospitalized for diverticular disease before baseline, developed a diverticular disease event within 1 year, or died or were lost to follow-up within 6 months. Additionally, individuals with missing data for five or more SNPs in the panel of PRS were also excluded (Supplementary Digital Content Figure 1, available at: http://links.lww.com/JS9/E758).

### Lifestyle factors and covariates

Lifestyle information and covariates were from self-reported records at baseline (2006–2010) in UKB, which were collected retrospectively at baseline. We systematically reviewed previously published literature to identify potential lifestyle factors associated with diverticular disease (Supplementary Digital Content Method, available at: http://links.lww.com/JS9/E758). A total of 17 potential lifestyle factors that have potential association with diverticular disease were included, and the definitions are described in detail in the Supplementary Digital Content Table 1, available at: http://links.lww.com/JS9/E758. These factors included smoking, alcohol, physical activity, sedentary time, sleep duration, sleeplessness/insomnia, tea intake, coffee intake, water intake, and healthy diet. A healthy diet score was calculated based on the adequate consumption of fruits, vegetables, whole grains, fish, and reduced consumption of refined grains, processed meats, and unprocessed red meats. A healthy diet included four or more of the above components. Covariates included age, sex, body mass index (BMI), ethnicity, and household income. Dummy indicators were introduced for missing data of continuous variables, while a dedicated “Missing” category was assigned for categorical variables with missing values.

### Polygenic risk score

The UKB provided direct genotyping information for around 805 000 single-nucleotide polymorphism (SNP) variants and imputed data for approximately 90 million variants, following rigorous quality control procedures[15]. The PRS used in this analysis was specifically derived from the UKB and externally validated by De Roo *et al*[8]. This PRS has demonstrated a strong correlation to both the incidence and severity of diverticulitis. Individuals in the highest decile of PRS were found to have a significantly higher likelihood of developing diverticulitis and experiencing severe complications compared to those in the bottom 50% of PRS scores[8]. For each individual in the cohort, the PRS was calculated using PLINK2 by multiplying the dosage of the risk-increasing alleles by their reported effect sizes and summing across all PRS loci. Scores were then standardized to a mean of zero and a standard deviation of one. To stratify genetic risk, participants were categorized into three groups – low, medium, and high PRS – according to PRS tertiles. Furthermore, a sensitivity analysis was performed using another PRS including 150 SNPs by Wu *et al*[14].

**HIGHLIGHTS**

- In this prospective cohort of 472 612 individuals, smoking, alcohol use, insomnia, sedentary behavior, and tea consumption increased diverticular disease risk, while healthy diets, coffee intake, and intermediate sleep duration reduced risk.
- Adopting healthier lifestyles lowers diverticular disease risk, with genetic background influencing specific lifestyle–disease relationships.
- Genetic predisposition may modify the effects of smoking, insomnia, and intake of refined grains/processed meats on diverticular disease risk.

### Outcome assessment

The primary outcome was incident diverticular disease of both small and large intestine. Participants’ medical records were tracked over time through linkage to electronic medical and other health-related records, which were updated approximately annually. Participants with diverticular disease were identified based on inpatient admissions with a corresponding International Classification of Diseases (ICD-10) code K57 in the primary/main position. The date of first in-patient diagnosis was used, which was recorded in either the primary or secondary position in the participant’s hospital inpatient records. In addition, analyses restricted to colonic diverticular disease were also performed.

To reduce the potential misclassification when the identified diverticular disease was not the primary reason for the corresponding admission, we conducted a sensitivity analysis focusing specifically on diverticulitis with perforation and abscess (as indicated by ICD codes K57.0, K57.2, K57.4, and K57.8), which is more likely to be identified as an incident event.

### Statistical analysis

Baseline characteristics were presented as sample size with percentages for categorical variables and medians with IQRs for continuous variables. The *χ*^2^ test and Mann–Whitney *U*-test were used to compare categorical variables and continuous variables, respectively. The follow-up period started from the date of recruitment to the date of first in-patient diagnosis of diverticular disease, death, lost to follow-up, or the end of current follow-up (31 October 2022, 31 August 2022, and 31 May 2022 in England, Scotland, and Wales, respectively), whichever came first. The Cox proportional hazards model was used to evaluate the risk of incident diverticular disease associated with each lifestyle factor, and hazard ratios (HRs) and 95% confidence intervals (CIs) were computed. Corresponding absolute risk differences were also present for the primary analyses.

Each lifestyle factor was analyzed separately in an univariable model (Model 1), model further adjusting for age, sex, and BMI (Model 2), and a fully adjusted model that additionally included ethnicity, household income, and PRS tertiles (Model 3). To explore the modifying effects of PRS on the relationship between lifestyle factors and diverticular disease, we conducted stratified analyses by PRS tertiles. A multiplicative interaction term was added to Model 3 to determine whether there was an interaction effect between each lifestyle factor and PRS. The interaction *P* value was calculated using a likelihood ratio test, comparing the model fit with and without the interaction term. A weighted lifestyle score was also constructed by multiplying the beta coefficients from Model 3 by 10 and rounding them to the nearest integer. The resulting scores were then divided into quartiles (Q1 to Q4) and were entered into the regression models[16].

A two-tailed *P*<0.05 was considered to be statistically significant in all analyses. To mitigate the impact of multiple testing, we applied a false-discovery-rate adjustment for the primary analysis. R software version 4.4.0 (The R Foundation for Statistical Computing, Vienna, Austria) was used for all statistical analyses. This study was been in line with the STROCSS criteria[17].

## Result

### Participants

A total of 472 612 participants were included in the current study. The median age of the study population was 58.0 years (IQR 50.0–64.0). Of all the participants, 215 999 (45.7%) were male, and 445 282 (94.2%) were white. This analysis encompassed 6 144 332 total person-years of follow-up. Over a median follow-up of 13.6 years (IQR 12.7–14.3), we identified 23 742 cases of diverticular disease, including 22 350 cases of colonic diverticular disease and 832 cases of diverticulitis with perforation and abscess. The proportions of cases by ICD-10 sub-codes are shown in the Supplementary Digital Content Table 2, available at: http://links.lww.com/JS9/E758.

The baseline characteristics were summarized for participants with and without diverticular disease (Supplementary Digital Content Table 3, available at: http://links.lww.com/JS9/E758). Compared with participants without diverticular disease, those who developed diverticular disease were more likely to be older [median age, 61 (54–65) vs 58 (50–63) years], women (58% vs 54.1%), white (96.4% vs 94.1%), and have a higher BMI. Of all the participants, 4.4% never drank alcohol and 54.8% never smoked, and both rates were lower in participants with diverticular disease. Moderate or vigorous physical activity was reported by 8.3% of the participants, with similar rates observed across the two groups. Participants with diverticular disease experienced more sleep-related disturbances. An ideal healthy diet was observed in 50.3% of participants without diverticular disease and 49.0% of those with diverticular disease. The correlation matrix of included lifestyle factors is shown in the Supplementary Digital Content Figure 2, available at: http://links.lww.com/JS9/E758 and no strong correlations existed among lifestyle factors, with coefficients ranging from −0.5 to 0.5.

### Individual lifestyle factors and diverticular disease

Overall, most of the lifestyle factors involved in this study were associated with the risk of diverticular disease, which were largely consistent in three models (Table 1). Corresponding absolute risk differences are shown in the Supplementary Digital Content Table 4, available at: http://links.lww.com/JS9/E758. In Model 3 adjusting for age, sex, BMI, ethnicity, household income, and PRS tertiles, smoking (former vs never: HR 1.14, 95% CI 1.11–1.18; current vs never: HR 1.21, 95% CI 1.16–1.26), alcohol consumption (former vs never: HR 1.13, 95% CI 1.03–1.24; moderate vs never: 1.04, 95% CI 0.98–1.11; heavy vs never: HR 1.12, 95% CI 1.05–1.20), frequent sleeplessness or insomnia (sometimes vs never: HR 1.12, 95% CI 1.08–1.16; usually vs never: HR 1.36, 95% CI 1.31–1.41), and more tea consumption (2–4 vs 0–1 cup: HR 1.10, 95% CI 1.06–1.13; over 5 vs 0–1 cup: HR 1.13, 95% CI 1.09–1.17) were associated with a higher risk of diverticular disease. Conversely, intermediate sleep duration (7–8 hours: HR 0.90, 95% CI 0.87–0.92) and coffee consumption (1–2 vs 0 cup: HR 0.93, 95% CI 0.9–0.96; over 3 vs 0 cup: HR 0.87, 95% CI 0.84–0.9) appeared to reduce the risk of diverticular disease. Participants with a healthy diet had a significantly lower risk of diverticular disease (HR 0.90, 95% CI 0.88–0.92). Specifically, participants who regularly consumed fruit (HR 0.89, 95% CI 0.87–0.91), vegetable (HR 0.92, 95% CI 0.89–0.96), wholegrain (HR 0.89, 95% CI 0.86–0.93), and fish (HR 0.96, 95% CI 0.94–0.99) were less likely to have diverticular disease. Avoiding processed meat (HR 0.93, 95% CI 0.91–0.96) and unprocessed red meat (HR 0.93, 95% CI 0.91–0.96) was found to be associated with reduced risk of diverticular disease. Considering the *P* value correction using a false-discovery-rate adjustment, the results of the majority of lifestyle factors remained consistent (Supplementary Digital Content Table 5, available at: http://links.lww.com/JS9/E758). Using a PRS based on 150 SNPs also yielded consistent results (Supplementary Digital Content Table 6, available at: http://links.lww.com/JS9/E758).

**Table 1:** Lifestyle factors and diverticular disease in three models

Variable	HR (95% CI)		
	Model 1^a^	Model 2^b^	Model 3^c^
Smoking			
Never	1	1	1
Former	1.27 (1.24–1.31)	1.16 (1.13–1.19)	1.14 (1.11–1.18)
Current	1.13 (1.08–1.18)	1.24 (1.18–1.29)	1.21 (1.16–1.26)
Alcohol			
Never	1	1	1
Former	1.18 (1.08–1.29)	1.22 (1.11–1.33)	1.13 (1.03–1.24)
Moderate	1.04 (0.97–1.11)	1.11 (1.04–1.18)	1.04 (0.98–1.11)
Heavy	1.08 (1.01–1.15)	1.19 (1.12–1.27)	1.12 (1.05–1.20)
Physical activity	1.01 (0.97–1.06)	1.02 (0.97–1.07)	1.01 (0.97–1.06)
Sedentary time (hours)			
0–2 h	1	1	1
2.1–4 h	1.15 (1.11, 1.19)	1.06 (1.02, 1.1)	1.05 (1.01, 1.09)
>4 h	1.36 (1.31, 1.41)	1.15 (1.11, 1.2)	1.14 (1.10, 1.18)
Intermediate sleep duration	0.87 (0.84–0.89)	0.90 (0.88–0.92)	0.90 (0.87–0.92)
Sleeplessness/insomnia			
Never/rarely	1	1	1
Sometimes	1.21 (1.17–1.26)	1.12 (1.08–1.16)	1.12 (1.08–1.16)
Usually	1.55 (1.50–1.61)	1.37 (1.32–1.42)	1.36 (1.31–1.41)
Tea (cups)			
0–1	1	1	1
2–4	1.14 (1.10–1.17)	1.09 (1.05–1.13)	1.10 (1.06–1.13)
≥5	1.19 (1.15–1.23)	1.14 (1.10–1.18)	1.13 (1.09–1.17)
Coffee (cups)			
0	1	1	1
1–2	0.99 (0.96–1.02)	0.93 (0.90–0.96)	0.93 (0.90–0.96)
≥3	0.91 (0.88–0.94)	0.88 (0.85–0.91)	0.87 (0.84–0.90)
Water intake (glasses)			
0–1	1	1	1
2–4	1.01 (0.98–1.04)	1.01 (0.99–1.04)	1.03 (1.00–1.06)
≥5	0.93 (0.9–0.97)	0.99 (0.95–1.03)	1.02 (0.98–1.06)
Healthy diet (≥4 scores)	0.95 (0.93–0.98)	0.89 (0.87–0.92)	0.90 (0.88–0.92)
Fruit^d^	0.96 (0.94–0.99)	0.88 (0.86–0.90)	0.89 (0.87–0.91)
Vegetable^d^	1.01 (0.97–1.04)	0.92 (0.89–0.95)	0.92 (0.89–0.96)
Wholegrain^d^	0.89 (0.86–0.93)	0.9 (0.86–0.94)	0.89 (0.86–0.93)
Refined grain^d^	0.97 (0.94–1.01)	0.97 (0.94–1.00)	0.98 (0.95–1.02)
Fish^d^	1.05 (1.02–1.07)	0.96 (0.94–0.98)	0.96 (0.94–0.99)
Processed meat^d^	0.97 (0.94–0.99)	0.92 (0.89–0.95)	0.93 (0.91–0.96)
Unprocessed red meat^d^	0.89 (0.87–0.91)	0.93 (0.91–0.96)	0.93 (0.91–0.96)

CI, confidence intervals; HR, hazard ratio.

^a^ Univariable model.

^b^ Model 2 adjusting for age, sex, and BMI.

^c^ Model 3 adjusting for age, sex, BMI, ethnicity, household income, and PRS tertiles.

^d^ Fruit ≥3 servings/day, vegetable ≥3 servings/day, wholegrain ≥3 servings/day, refined grain ≤1.5 servings/day, fish ≥2 servings/week, processed meat ≤1 serving/week, unprocessed red meat ≤1.5 servings/week.

For diverticular disease of the colon, the results were consistent. Smoking (former vs never: HR 1.15, 95% CI 1.11–1.18; current vs never: HR 1.20, 95% CI 1.14–1.25), alcohol consumption (former vs never: HR 1.12, 95% CI 1.02–1.23; moderate vs never: 1.04, 95% CI 0.97–1.11; heavy vs never: HR 1.13, 95% CI 1.05–1.21), frequent sleeplessness or insomnia (sometimes vs never: HR 1.12, 95% CI 1.08–1.16; usually vs never: HR 1.34, 95% CI 1.29–1.40), and more tea consumption (2–4 vs 0–1 cup (s): HR 1.10, 95% CI 1.06–1.14; over 5 vs 0–1 cup: HR 1.13, 95% CI 1.09–1.17) remained risk factors (Table 2). Coffee intake and healthy diet habits may play a protective role.

**Table 2:** Lifestyle factors and colonic diverticular disease or complicated diverticulitis

Variable	Colonic diverticular disease	Diverticulitis with perforation and abscess
	HR (95% CI)^a^	HR (95% CI)^a^
Smoking		
Never	1	1
Former	1.15 (1.11–1.18)	1.30 (1.12–1.52)
Current	1.20 (1.14–1.25)	2.48 (2.05–3.00)
Alcohol		
Never		
Former	1.12 (1.02–1.23)	1.46 (0.94–2.28)
Moderate	1.04 (0.97–1.11)	0.93 (0.65–1.31)
Heavy	1.13 (1.05–1.21)	1.11 (0.78–1.60)
Physical activity	1.01 (0.97–1.06)	1.16 (0.91–1.47)
Sedentary time (hours)		
0–2 h	1	1
2.1–4 h	1.04 (1.01–1.08)	1.01 (0.84–1.22)
>4 h	1.13 (1.08–1.17)	1.29 (1.07–1.57)
Intermediate sleep duration	0.90 (0.88–0.93)	0.89 (0.77–1.03)
Sleeplessness/insomnia		
Never/rarely	1	1
Sometimes	1.12 (1.08–1.16)	1.05 (0.88–1.26)
Usually	1.34 (1.29–1.40)	1.31 (1.08–1.59)
Tea (cups)		
0–1	1	1
2–4	1.10 (1.06–1.14)	1.02 (0.86–1.21)
≥5	1.13 (1.09–1.17)	1.00 (0.84–1.21)
Coffee (cups)		
0	1	1
1–2	0.93 (0.90–0.96)	0.93 (0.78–1.10)
≥3	0.87 (0.84–0.90)	0.98 (0.83–1.17)
Water intake (glasses)		
0–1	1	1
2–4	1.03 (1.00–1.06)	0.99 (0.85–1.16)
≥5	1.02 (0.98–1.06)	0.98 (0.79–1.21)
Healthy diet (≥4 scores)	0.90 (0.88–0.92)	0.85 (0.74–0.97)
Fruit^b^	0.89 (0.87–0.92)	0.79 (0.69–0.90)
Vegetable^b^	0.93 (0.90–0.96)	0.86 (0.72–1.03)
Wholegrain^b^	0.90 (0.86–0.94)	0.78 (0.61–0.98)
Refined grain^b^	0.97 (0.94–1.01)	1.12 (0.94–1.35)
Fish^b^	0.96 (0.94–0.99)	0.98 (0.85–1.12)
Processed meat^b^	0.93 (0.91–0.96)	0.95 (0.81–1.10)
Unprocessed red meat^b^	0.94 (0.91–0.96)	0.98 (0.85–1.12)

CI, confidence intervals; HR, hazard ratio.

^a^ Adjusting for age, sex, BMI, ethnicity, household income, and PRS tertiles.

^b^ Fruit ≥3 servings/day, vegetable ≥3 servings/day, wholegrain ≥3 servings/day, refined grain ≤1.5 servings/day, fish ≥2 servings/week, processed meat ≤1 serving/week, unprocessed red meat ≤1.5 servings/week.

For diverticulitis with perforation and abscess, smoking (former vs never: HR 1.30, 95% CI 1.12–1.52; current vs never: HR 2.48, 95% CI 2.05–3.00) and frequent insomnia (usually vs never: HR 1.31, 95% CI 1.08–1.59) remained risk factors. In addition, a healthy diet (HR 0.85, 95% CI 0.74–0.97) was also significantly associated with a lower incidence of diverticulitis with perforation and abscess, particularly diets rich in fruit (HR 0.79, 95% CI 0.69–0.90) and wholegrain (HR 0.78, 95% CI 0.61–0.98) (Table 2).

### Stratified analyses by PRS

Current smoking was associated with a higher risk of diverticular disease, particularly in low and high PRS tertiles (*P* for interaction: 0.030). Sleeplessness or insomnia was associated with a higher risk across PRS tertiles, where the highest risk was observed in individuals within the high tertile (*P* for interaction: 0.031). Tea intake and PRS also showed a significant interaction, where individuals who consumed ≥ 5 cups of tea per day and PRS in the intermediate tertile had the highest risk (*P* for interaction: 0.044). The interaction was also observed between PRS and consumption of refined grain and unprocessed red meats (*P* for interaction: 0.014 and 0.023), which were associated with a lower risk across PRS tertiles (Table 3). The results were consistent when stratifying by the tertiles of PRS of 150 SNPs (Supplementary Digital Content Table 6, available at: http://links.lww.com/JS9/E758).

**Table 3:** Lifestyle factors and risk of diverticular disease according to polygenic risk score

Variable	HR (95% CI) for diverticular disease^a^			*P* for interaction^b^
	Tertile 1 of PRS	Tertile 2 of PRS	Tertile 3 of PRS	
Smoking				0.030
Never	1	1	1	
Former	1.18 (1.12–1.25)	1.08 (1.03–1.14)	1.17 (1.12–1.23)	
Current	1.23 (1.13–1.34)	1.18 (1.09–1.27)	1.22 (1.14–1.31)	
Alcohol				0.376
Never	1	1	1	
Former	1.17 (0.98–1.40)	1.13 (0.96–1.32)	1.11 (0.96–1.28)	
Moderate	1.04 (0.91–1.18)	1.05 (0.94–1.18)	1.04 (0.94–1.15)	
Heavy	1.16 (1.01–1.32)	1.12 (0.99–1.26)	1.10 (0.99–1.23)	
Physical activity	1.02 (0.93–1.11)	1.01 (0.93–1.1)	1.01 (0.94–1.09)	0.151
Sedentary time (hours)				0.055
0–2 h	1	1	1	
2.1–4 h	1.02 (0.95–1.09)	1.03 (0.97–1.09)	1.09 (1.03–1.15)	
>4 h	1.16 (1.08–1.25)	1.09 (1.02–1.17)	1.16 (1.10–1.24)	
Intermediate sleep duration	0.94 (0.89–0.99)	0.90 (0.86–0.94)	0.87 (0.83–0.91)	0.180
Sleeplessness or insomnia				0.031
Never/rarely	1	1	1	
Sometimes	1.09 (1.02–1.17)	1.13 (1.07–1.21)	1.13 (1.06–1.19)	
Usually	1.25 (1.16–1.34)	1.37 (1.28–1.46)	1.43 (1.35–1.52)	
Tea (cups)				0.044
0–1	1	1	1	
2–4	1.08 (1.02–1.15)	1.10 (1.04–1.17)	1.10 (1.05–1.16)	
≥5	1.09 (1.02–1.17)	1.18 (1.11–1.25)	1.12 (1.06–1.19)	
Coffee (cups)				0.559
0	1	1	1	
1–2	0.95 (0.90–1.01)	0.92 (0.87–0.97)	0.92 (0.88–0.96)	
≥3	0.89 (0.83–0.95)	0.86 (0.81–0.91)	0.87 (0.82–0.91)	
Water intake (glasses)				0.154
0–1	1	1	1	
2–4	1.07 (1.01–1.14)	0.98 (0.94–1.03)	1.03 (0.98–1.08)	
≥5	1.08 (1.00–1.17)	0.96 (0.89–1.03)	1.03 (0.97–1.10)	
Healthy diet (≥4 scores)	0.88 (0.83–0.92)	0.90 (0.86–0.94)	0.92 (0.88–0.95)	0.328
Fruit^c^	0.86 (0.82–0.90)	0.91 (0.87–0.95)	0.89 (0.86–0.93)	0.150
Vegetable^c^	0.91 (0.85–0.97)	0.93 (0.88–0.99)	0.92 (0.88–0.98)	0.660
Wholegrain^c^	0.87 (0.80–0.94)	0.90 (0.84–0.97)	0.90 (0.84–0.96)	0.148
Refined grain^c^	0.99 (0.93–1.06)	0.98 (0.92–1.03)	0.98 (0.93–1.04)	0.014
Fish^c^	0.95 (0.90–0.997)	0.97 (0.93–1.01)	0.96 (0.92–0.999)	0.725
Processed meat^c^	0.89 (0.84–0.94)	0.95 (0.9–0.997)	0.94 (0.90–0.99)	0.057
Unprocessed red meat^c^	0.89 (0.85–0.94)	0.94 (0.9–0.98)	0.96 (0.92–0.998)	0.023

CI, confidence intervals; HR, hazard ratio; PRS, polygenic risk score.

^a^ Adjusting for age, sex, BMI, ethnicity, and household income.

^b^ Likelihood ratio test; All *P* values are non-significant after false-discovery-rate adjustment.

^c^ Fruit ≥3 servings/day, vegetable ≥3 servings/day, wholegrain ≥3 servings/day, refined grain ≤1.5 servings/day, fish ≥2 servings/week, processed meat ≤1 serving/week, unprocessed red meat ≤1.5 servings/week.

For colonic diverticular disease, additional interaction effects with genetic susceptibility were observed for less intake of processed meat, while no significant interactions were found for sleeplessness or insomnia and more tea intake (Supplementary Digital Content Table 7, available at: http://links.lww.com/JS9/E758). For diverticulitis with perforation and abscess, the effects of alcohol consumption, physical activity, sleep duration, and the consumption of fish on its risk appeared to be influenced by one’s genetic predisposition (Supplementary Digital Content Table 8, available at: http://links.lww.com/JS9/E758).

### Weighted lifestyle score and risk of diverticular disease

We found that patients with higher weighted lifestyle score (Supplementary Digital Content Table 9, available at: http://links.lww.com/JS9/E758) were associated with a higher risk of diverticular disease (Q2 vs Q1: HR 1.19, 95% CI 1.14–1.25; Q3 vs Q1: HR 1.32, 95% CI 1.25–1.39; Q4 vs Q1: HR 1.56, 95% CI 1.49–1.64), colonic diverticular disease (Q2 vs Q1: HR 1.19, 95% CI 1.13–1.25; Q3 vs Q1: HR 1.31, 95% CI 1.25–1.39; Q4 vs Q1: HR 1.55, 95% CI 1.48–1.62), and diverticulitis with perforation and abscess (Q2 vs Q1: HR 1.40, 95% CI 1.05–1.85; Q3 vs Q1: HR 1.24, 95% CI 1.24–2.24; Q4 vs Q1: HR 2.08, 95% CI 1.59–2.73, Table 4). In addition, higher genetic risk appears to slightly exacerbate this risk.

**Table 4:** Weighted lifestyle score and risk of diverticular disease

Weighted lifestyle score	HR (95% CI)			
	Model 3^a^	Tertile 1 of PRS^b^	Tertile 2 of PRS^b^	Tertile 3 of PRS^b^
**Diverticular disease**				
Q1	1	1	1	1
Q2	1.19 (1.14–1.25)	1.14 (1.04–1.25)	1.19 (1.09–1.29)	1.23 (1.14–1.32)
Q3	1.32 (1.25–1.39)	1.29 (1.16–1.42)	1.32 (1.21–1.45)	1.33 (1.23–1.45)
Q4	1.56 (1.49–1.64)	1.50 (1.38–1.65)	1.53 (1.41–1.66)	1.64 (1.52–1.76)
**Colonic diverticular disease**				
Q1	1	1	1	1
Q2	1.19 (1.13–1.25)	1.15 (1.05–1.27)	1.17 (1.08–1.28)	1.22 (1.12–1.32)
Q3	1.31 (1.25–1.39)	1.33 (1.2–1.48)	1.29 (1.18–1.42)	1.32 (1.22–1.44)
Q4	1.55 (1.48–1.62)	1.52 (1.39–1.67)	1.50 (1.38–1.62)	1.61 (1.49–1.74)
**Diverticulitis with perforation and abscess**				
Q1	1	1	1	1
Q2	1.40 (1.05–1.85)	1.32 (0.74–2.35)	1.04 (0.63–1.72)	1.73 (1.13–2.65)
Q3	1.67 (1.24–2.24)	1.66 (0.91–3.04)	1.63 (0.98–2.73)	1.70 (1.07–2.69)
Q4	2.08 (1.59–2.73)	2.01 (1.16–3.47)	1.88 (1.18–3.00)	2.29 (1.51–3.47)

CIs, confidence intervals; HR, hazard ratio; PRS, polygenic risk score; Q, quartile.

^a^ Model 3 adjusting for age, sex, BMI, ethnicity, household income, and PRS tertiles.

^b^ Adjusting for age, sex, BMI, ethnicity, and household income.

## Discussion

In this large prospective cohort study, we found that adopting an optimal lifestyle was associated with a lower risk of developing diverticular disease. Across all genetic backgrounds, lifestyle factors such as smoking cessation, reduced alcohol intake, good sleep habit, high coffee consumption, low tea consumption, and a healthy diet – characterized by higher consumption of fruits, vegetables, whole grains, and fish, along with lower intake of processed meats and unprocessed red meat – were linked to a lower risk of colonic diverticular disease. In addition, the impact of certain lifestyle factors may be modified by genetic predisposition.

Our study supports the well-established association between smoking and diverticular disease, particularly complicated diverticulitis^[1,7,18]^. A Nurses’ Health Study II (NHS II) analysis by Gunby *et al* found that current and former smokers have a similar higher risk of diverticulitis than never smokers[7]. Our study found current smokers have a 1.2 times higher risk of diverticular disease and a 2.48 times higher risk of complicated diverticulitis, aligning with the NHS II’s findings that the smoking-diverticulitis association was strengthened in cases needing surgical management[7]. Notably, compared with current smokers, our study found that former smokers were at a lower risk, especially for complicated diverticulitis, underscoring the long-term benefits of smoking cessation. Smoking-induced chronic inflammation, reflected by elevated levels of inflammatory biomarkers[19], may contribute to the heightened risk of diverticulitis[6], suggesting that quitting smoking can reduce the risk, even after prolonged exposure.

Alcohol consumption has been less studied in relation to diverticulitis. Our findings highlighted an increased risk for both heavy drinkers and former drinkers, particularly for complicated diverticulitis. Moderate drinkers, however, did not exhibit a similar increase in risk. Our results were consistent with those of Gunby *et al* in the NHS II, which showed that higher alcohol intake in women, particularly consuming more than 30 g per day, was associated with an increased risk of diverticulitis[7]. Although the precise mechanisms remain unclear, alcohol’s potential effects on diverticulitis may involve reduced prostaglandin synthesis, which plays a role in inflammatory processes[20].

Physical activity has been linked to a lower risk of diverticulitis[21], although our overall analysis did not reach statistical significance. Notably, we observed a positive association between moderate to vigorous physical activity and the risk of complicated diverticulitis in participants with intermediate PRS, suggesting that genetic factors may modulate this relationship. Previous studies, such as those from the Health Professionals Follow-up Study and a Swedish cohort, have shown that vigorous exercise and higher levels of physical activity can reduce the risk of diverticulitis and diverticular bleeding^[22,23]^. Additionally, in line with a previous study, our study found that sedentary behavior was linked to an increased risk of diverticular disease[24]. Physical activity promotes intestinal motility and reduces transit time[25], which may lower intra-colonic pressure and thus prevent diverticular complications. Furthermore, exercise influences hormones that regulate gastrointestinal motility, providing additional protective effects[26].

The role of sleep quality in diverticular disease has been less explored. Our study indicated that intermediate sleep duration was associated with a reduced risk of diverticular disease. A similar protective effect was observed for complicated diverticulitis among those with intermediate PRS. Conversely, frequent insomnia or sleeplessness was associated with an increased risk. While a previous study found a link between obstructive sleep apnea and diverticular bleeding, the relationship between sleep quality and diverticulitis remains unclear[27]. Nonetheless, disturbed sleep has been associated with other gastrointestinal disorders, such as irritable bowel syndrome and constipation, potentially through mechanisms involving the brain–gut axis^[28,29]^.

The evidence on the effects of tea and coffee consumption on diverticular disease is also limited. Our findings indicated that tea consumption may increase the risk of diverticular disease. While prior studies have not demonstrated a clear association between coffee consumption and diverticular disease^[30,31]^, our study suggested an inverse relationship between coffee intake and both diverticular disease and complicated diverticulitis. These seemingly paradoxical opposing effects of tea and coffee consumption could be partially explained by coffee’s anti-inflammatory properties, which were linked to bioactive compounds (e.g., niacin) rather than caffeine alone, as evidenced in studies reporting reduced inflammatory biomarkers^[32,33]^. In contrast, the opposite effect of tea on diverticular outcomes might stem from variations in polyphenol content, preparation methods (such as water temperature and additives), concomitant water drinking, and gender-specific factors[34]. In addition, certain lifestyle confounders – such as smoking, which often co-occurs with tea drinking in various cultures – may partially account for the observed increased risk of diverticular disease among tea drinkers. Differences in caffeine metabolism or bioactive compound extraction further complicate these associations, underscoring the need for further research into the underlying mechanisms and population-specific influences[35].

Dietary factors play a critical role in the pathogenesis of diverticulitis. Our study found that higher consumption of fruits and whole grains was associated with a lower risk of both diverticular disease and complicated diverticulitis, whereas vegetable consumption showed no such association. The protective effects of fruits and whole grains are likely mediated by their high fiber content, which has been well documented[11]. Consistent with previous research, fiber from fruits and cereals – but not vegetables – was inversely associated with diverticulitis[11]. We further observed that higher fish consumption was associated with a lower risk of diverticular disease, possibly reflecting the anti-inflammatory properties of omega-3 fatty acids[36], although one study found that fish alone had no effect unless substituted for unprocessed red meat[12]. Consistent with previous studies, we also revealed that lower intake of both processed and unprocessed red meats was associated with a reduced risk of diverticular disease^[12,37]^.

Beyond individual foods, overall dietary patterns are essential, and a healthy diet was associated with a reduced risk for incident diverticulitis^[38,39]^. Strate *et al* reported that a Western dietary pattern – rich in red and processed meats, refined grains, sweets, and high-fat dairy – increased diverticulitis risk, while a prudent diet – rich in fruits, vegetables, whole grains, legumes, poultry, and fish – was linked to a reduced risk[39]. Although our study assessed a general dietary score rather than adherence to specific patterns (e.g., Mediterranean diet), evidence suggests that components of such healthy diets support favorable microbial diversity, enhance colonic fermentation, increase short–chain fatty acid production, and preserve mucosal integrity – collectively reducing the propensity for diverticular formation^[38,40]^. Conversely, alterations in inflammation-related microbial and metabolic profiles may exacerbate mucosal inflammation and elevate intraluminal pressure, facilitating both the development and progression of diverticula. Indeed, patients with diverticular disease frequently exhibit diminished microbial diversity, lower levels of fiber-fermenting bacteria, and upregulated pro-inflammatory pathways^[40,41]^. Overall, our findings reinforce the importance of a healthy diet targeted interventions – ranging from prebiotics and probiotics to dietary modification aimed at restoring microbial balance – in preventing and managing diverticulitis, especially in patients who develop complications or require surgical intervention^[42,43]^.

Genomic studies have increasingly highlighted the role of genetic susceptibility in diverticular disease^[10,13]^. Our study suggested that an individual’s genetic makeup may modulate associations of lifestyle factors with diverticular disease, although the directionality of these interactions varied. For instance, the risks associated with sleeplessness or insomnia and low unprocessed red meat intake were amplified among individuals with higher PRS, while the risk of smoking decreased at intermediate PRS. Conversely, the risk of tea consumption was elevated at intermediate PRS. These findings suggest that integrating genetic risk assessments with lifestyle interventions could enhance prevention. However, most interactions may lack clear biological plausibility and do not remain significant after multiple testing corrections; therefore, they warrant cautious interpretation.

The strengths of our study include the large-scale prospective cohort design, with long-term follow-up, comprehensive collection of lifestyle information, and thorough assessment of diverticular disease and complicated diverticulitis. Moreover, the PRS used in this study was robust and externally validated previously. However, there are several limitations in this study. First, the diagnosis of diverticular disease was extracted based on ICD-10 codes extracted from the inpatient records, which may have resulted in misclassification, particularly for uncomplicated diverticulitis. We attempted to mitigate this by focusing on complicated diverticulitis with perforation and abscess separately. Second, lifestyle assessments at baseline (e.g., alcohol intake, smoking status) are susceptible to non-differential misclassification, particularly given the time-varying nature of these behaviors. Such misclassification would likely attenuate observed HRs toward the null, potentially obscuring true associations. Additionally, self-reported data may be prone to measurement errors. Third, the UKB cohort is predominantly White British; our findings may not generalize to underrepresented populations (e.g., Africans or South Asians) with different susceptibility and background prevalence. Future studies in diverse cohorts will determine whether lifestyle associations observed here hold true in other regions or populations. Last, the PRS used in this study was developed based on UKB data, which may introduce overfitting when applying it back to its training data. We assessed the overfitting by splitting the dataset into training (70%) and testing (30%) subsets. The Concordance index was 0.626 and 0.628 for training and testing subsets, respectively. The genetic effect estimates (HR per unit increase in PRS) were consistent between subsets (training HR: 2.04, 95% CI 1.94–2.15; testing HR: 1.98, 95% CI: 1.83–2.15). These results suggested minimal overfitting.

In conclusion, smoking, alcohol consumption, frequent insomnia, sedentary behavior, and high tea consumption were significantly associated with an increased risk of diverticular disease. Conversely, adopting a healthy lifestyle – including intermediate sleep duration, higher coffee consumption, and a diet rich in fruits, vegetables, whole grains, and fish, while reducing intake of processed meats and unprocessed red meats – was associated with a lower risk of diverticular disease. The impact of these lifestyle factors may be modified by genetic susceptibility, suggesting that personalized prevention strategies that combine genetic risk assessment with lifestyle modifications could help reduce the burden of diverticular disease.

## Data Availability

Data can be available on request from the UK Biobank (www.ukbiobank.ac.uk/).

## Research registration unique identifying number (UIN)

Not applicable. This is a secondary analysis of the UK Biobank.
